# Supplementary material for: Membrane lipid nanodomains modulate HCN pacemaker channels in nociceptor DRG neurons
Source: Nat Commun. 2024 Nov 15;15:9898. doi: 10.1038/s41467-024-54053-z (PMC11568329; doi:10.1038/s41467-024-54053-z)
Supplement: Supplementary file 2 — Reporting Summary [file 41467_2024_54053_MOESM2_ESM.pdf]

nature portfolio

Corresponding author(s): Gucan Dai
Last updated by author(s): Oct 7, 2024

Reporting Summary

Nature Portfolio wishes to improve the reproducibility of the work that we publish. This form provides structure for consistency and transparency in reporting. For further information on Nature Portfolio policies, see our Editorial Policies and the Editorial Policy Checklist.

Statistics

For all statistical analyses, confirm that the following items are present in the figure legend, table legend, main text, or Methods section.

n/a	Confirmed	
☐	☒	The exact sample size (n) for each experimental group/condition, given as a discrete number and unit of measurement
☐	☒	A statement on whether measurements were taken from distinct samples or whether the same sample was measured repeatedly
☐	☒	The statistical test(s) used AND whether they are one- or two-sided<br>*Only common tests should be described solely by name; describe more complex techniques in the Methods section.*
☒	☐	A description of all covariates tested
☒	☐	A description of any assumptions or corrections, such as tests of normality and adjustment for multiple comparisons
☐	☒	A full description of the statistical parameters including central tendency (e.g. means) or other basic estimates (e.g. regression coefficient) AND variation (e.g. standard deviation) or associated estimates of uncertainty (e.g. confidence intervals)
☐	☒	For null hypothesis testing, the test statistic (e.g. F, t, r) with confidence intervals, effect sizes, degrees of freedom and P value noted<br>*Give P values as exact values whenever suitable.*
☒	☐	For Bayesian analysis, information on the choice of priors and Markov chain Monte Carlo settings
☒	☐	For hierarchical and complex designs, identification of the appropriate level for tests and full reporting of outcomes
☒	☐	Estimates of effect sizes (e.g. Cohen's d, Pearson's r), indicating how they were calculated

Our web collection on statistics for biologists contains articles on many of the points above.

Software and code

Policy information about availability of computer code

Data collection	PatchMaster (HEKA Elektronik), INFINITY ANALYZE and CAPTURE (TELEDYNE LUMENERA), VistaVision (ISS, Inc.)
Data analysis	Igor Pro 9, ImageJ 1.49, VistaVision (ISS, Inc.)

For manuscripts utilizing custom algorithms or software that are central to the research but not yet described in published literature, software must be made available to editors and reviewers. We strongly encourage code deposition in a community repository (e.g. GitHub). See the Nature Portfolio guidelines for submitting code & software for further information.

Data

Policy information about availability of data

All manuscripts must include a data availability statement. This statement should provide the following information, where applicable:

- Accession codes, unique identifiers, or web links for publicly available datasets
- A description of any restrictions on data availability
- For clinical datasets or third party data, please ensure that the statement adheres to our policy

Data availability statement is provided with the paper. All Source Data sets for Figs. 1-8, Fig. S1-S11 are included as Source Data file. Other data are available from the corresponding author upon request. PDB accession code used in the Fig. 8 has been referred to in the data availability statement.

Research involving human participants, their data, or biological material

Policy information about studies with human participants or human data. See also policy information about sex, gender (identity/presentation), and sexual orientation and race, ethnicity and racism.

Reporting on sex and gender	N/A
Reporting on race, ethnicity, or other socially relevant groupings	N/A
Population characteristics	N/A
Recruitment	N/A
Ethics oversight	N/A

Note that full information on the approval of the study protocol must also be provided in the manuscript.

Field-specific reporting

Please select the one below that is the best fit for your research. If you are not sure, read the appropriate sections before making your selection.

☒ Life sciences ☐ Behavioural & social sciences ☐ Ecological, evolutionary & environmental sciences

For a reference copy of the document with all sections, see nature.com/documents/nr-reporting-summary-flat.pdf

Life sciences study design

All studies must disclose on these points even when the disclosure is negative.

Sample size	Sample size was based on previously published results using patch-clamp recordings of cell lines and primary cells, rat behavior tests, or applying ensemble tmFRET that generated sufficient statistical power, and also with the consideration of technical aspect of applying dual stop-codon suppression for genetic code expansion on imaging membrane ion channels.
Data exclusions	No successful recordings or imaging data were excluded from the analysis.
Replication	All experiments were repeated and confirmed to be reproducible. Key experiments were conducted with at least two independent cell transfections or surgical preparations from animals. The specific number of independent replicates is provided in the figure legends.
Randomization	The experiments were conducted randomly, i.e. control experiment imaged first and then treatment group, or vice versa
Blinding	The investigators maintained awareness of the experimental conditions throughout the data acquisition and analysis process, and the investigators were unbiased in both outcome assessment and conclusion drawing.

Reporting for specific materials, systems and methods

We require information from authors about some types of materials, experimental systems and methods used in many studies. Here, indicate whether each material, system or method listed is relevant to your study. If you are not sure if a list item applies to your research, read the appropriate section before selecting a response.

Materials & experimental systems

n/a	Involved in the study	
☒	☐	Antibodies
☐	☒	Eukaryotic cell lines
☒	☐	Palaeontology and archaeology
☐	☒	Animals and other organisms
☒	☐	Clinical data
☒	☐	Dual use research of concern
☒	☐	Plants

Methods

n/a	Involved in the study	
☒	☐	ChIP-seq
☒	☐	Flow cytometry
☒	☐	MRI-based neuroimaging

Eukaryotic cell lines

Policy information about cell lines and Sex and Gender in Research

Cell line source(s)	Established tsA 201 cells, embryonal kidney, SV40 transformed, purchased from MilliporeSigma, catalog number 96121229. H9C2 rat cardiomyocyte cells were obtained from ATCC (CRL-1446).
Authentication	Cell lines were authenticated and confirmed using STR profiling by Millipore Sigma, no further authentication was performed
Mycoplasma contamination	MycoFluor™ Mycoplasma Detection Kit (Invitrogen) was used to test for contamination and the result was negative.
Commonly misidentified lines (See ICLAC register)	No misidentified cell lines. No other cell lines used in the lab.

Animals and other research organisms

Policy information about studies involving animals; ARRIVE guidelines recommended for reporting animal research, and Sex and Gender in Research

Laboratory animals	Mice used for cardiac pacemaker, primary hepatocyte, and dermal fibroblast isolation were all C57BL/6J strain and sourced from in house breeding colony. Both male and female mice were used for cell isolations, all 8-20 weeks of age. Adult Sprague-Dawley rats, including pathogen-free female 100 g, male 250 g (5-7 weeks of age) were sourced from Envigo (Placentia, CA)
Wild animals	N/A
Reporting on sex	For rat DRG neuron studies, equal groups were used for each sex when feasible to detect potential sex differences. For primary cells from mouse, cell isolations were performed in both male and female. The mouse studies were not powered to detect sex differences, given the assumption that sex would not significantly impact the measured outcomes. Future work could explore potential sex differences in greater detail.
Field-collected samples	N/A
Ethics oversight	Animal procedures for both mice and rats were approved by the IACUC of Saint Louis University.

Note that full information on the approval of the study protocol must also be provided in the manuscript.
